# Supplementary material for: Thermodynamic signatures of the field-induced states of graphite
Source: Nat Commun. 2017 Nov 7;8:1337. doi: 10.1038/s41467-017-01394-7 (PMC5677099; doi:10.1038/s41467-017-01394-7)
Supplement: Supplementary file 1 — Supplementary Information [file 41467_2017_1394_MOESM1_ESM.pdf]

Supplementary Note 1 Ultrasound measurements

We report on Supplementary Figure 1 panel a and b, the change in the sound velocity $\Delta v/v_0$, and in the sound attenuation $\Delta\alpha(\omega)$, up to 57 T for temperatures ranging from 1.4K to 15K. Low field quantum oscillations are best seen in panel c and are discussed below. Above 7.5 T, all the holes and the electrons are in the (n=0,±) Landau levels, the sound velocity increases linearly with the magnetic field and the sound attenuation is almost field independent up to the entrance in the field-induced state where dramatic changes occur as discussed in the main text. Panel c shows the sound velocity change at 74 and 36 MHz, i.e. in the thermodynamic limit where $2\pi f\tau_0 << 1$.

As previously reported by Inoue et al. [2] we find that thermal cycling damages the ultrasound signal quality. The α, β and δ transition fields are observed at the same magnetic field in all samples, suggesting those field scales are not subject to defects in the sample. In contrast, we find that the γ and δ^* transitions vary from one sample to another suggesting their dynamics are affected by sample quality and history.

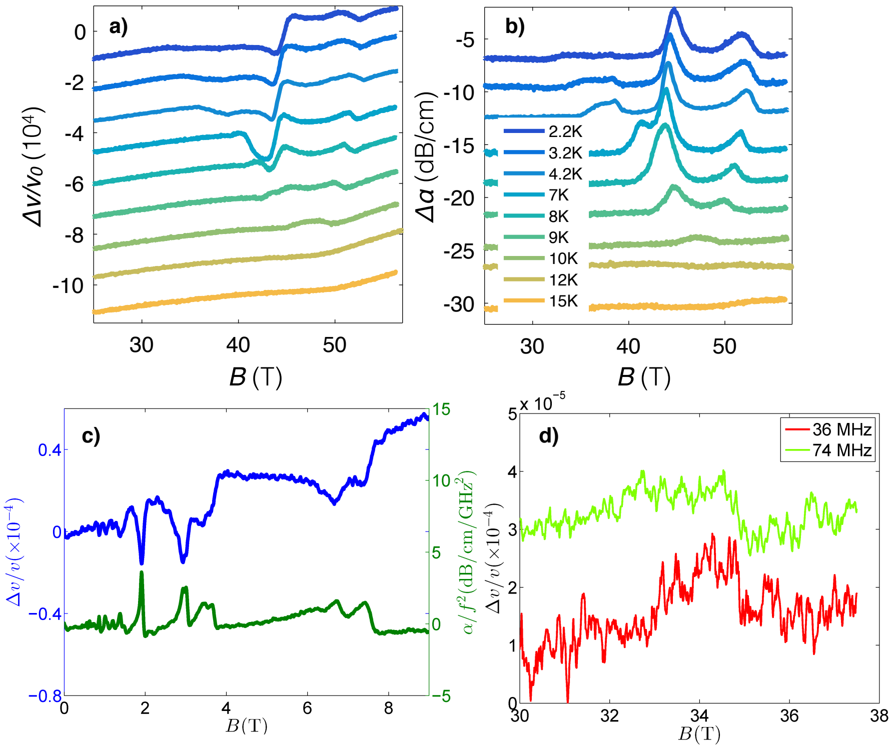

Supplementary Figure 1 Ultrasound properties of graphite : a) $\frac{\Delta v}{v_0}$ and b) $\Delta\alpha(\omega)$ as a function of the magnetic field for f=255MHz and temperature ranging from 1.4 to 15K. Curves are shifted vertically for clarity. c) Comparison of low field quantum oscillations in the sound attenuation (green) and velocity (blue) at $T = 0.76$ K and $f = 227$ MHz in HOPG. d) Jump in the sound velocity at the lowest frequencies studied $f = 36$ (in red) and 74 MHz (in green) at $T = 4.2$ K.

Low magnetic field oscillatory ultrasound velocity and attenuation

Since the works of Gurevich [1] giant quantum oscillations are expected to form in the acoustical absorption of semi-metals in presence of a magnetic field. These oscillations are the result of the surf riding condition [5]. In a simple picture, for a sound wave propagating parallel to the magnetic field ($\mathbf{q}//\mathbf{B}$), when the magnetic field is such that the sound velocity v_0 becomes equal to v_F^z (the component of the Fermi velocity along the field) the electrons are able to extract more energy from the wave than at other field. Thus when the Fermi energy is crossing a Landau level, the ultrasound absorption increases. These effects have been intensively studied in the case of bismuth, but poorly and only up to 2T in the case of graphite [2]. In Supplementary Figure 1 panel c), we show low field quantum oscillations up to 9 T. The spectrum observed here reproduces early work of graphite [2]. The attenuation peaks have

a shape reminiscent of the Nernst effect oscillations [6], mimicking the change in the density of state at the Fermi level. LL crossing corresponds to an absorption maximum and to a sound velocity local minimum.

Supplementary Note 2 Resistivity measurements

Standard resistivity measurements were performed on the same HOPG samples as used for the ultrasound measurements and on additional Kish graphite samples. Transport experiments have been conducted in static magnetic fields at the LNCMI-Grenoble up to 32T and down to 0.4K, and in pulsed magnetic field at the LNCMI-Toulouse. We report on Supplementary Figure 2 the transverse (R_{xx}) and the longitudinal (R_{zz}) magnetoresistance. In R_{xx} a first transition characterized by sharp increase is followed by a plateau, attributed to a second transition [3, 4]. The onset magnetic field in R_{zz} has been evaluated by taking the field at which R_{zz} becomes 30% larger (the typical variation of R_{xx} in the field-induced state) than $R_{zz}(B_0)$ where B_0 is the onset field in R_{xx}. Above this field the increase in R_{zz} cannot be attributed to a contamination of R_{zz} by in-plane transport coefficient R_{xx}. This procedure gives the onset field scale plotted as black circles in Supplementary Figure 2 and reported as black diamonds found at the β transition in Fig. 2 of the main text. The error bars found in Fig. 2 of the main text have been estimated by measurements of five different samples.

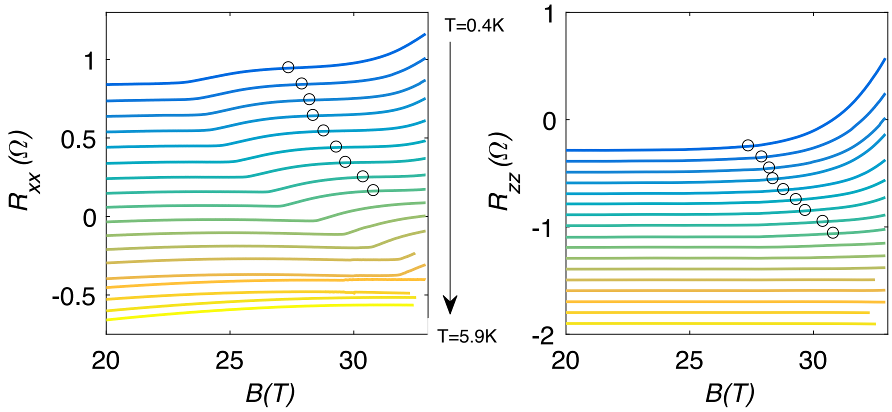

Supplementary Figure 2 Comparison of R_{xx} and R_{zz} magnetoresistance on Kish graphite. The magnetic field is applied along the c-axis. The black points correspond to the onset of the field-induced state in R_{zz} (see text for the details).

Supplementary Note 3 Order parameter relaxation rate

The Landau-Khalatnikov analysis of the α-transition performed in Supplementary figure 3 yields a low relaxation rate of the order parameter varying as $f_c = f_0\varepsilon \approx 8 \times 10^8\varepsilon$ Hz at 36.1 T, with ε the reduced temperature. f_0 can also be determined using Fig. 5c of the main text. At 5 K we get $f_0 \approx 3$ GHz, slightly higher than the value determined from the temperature dependence. This indicates that the fluctuation spectrum is different when the ground state of the system is controlled with a non-thermal parameter. Nonetheless, f_0 found here in graphite is low compared to what is usually observed at a phase transition. In magnetic systems such as Ni[7] or $RbMnF_3$[8] it is found that $f_c = 2 \times 10^{14}\varepsilon^{1.8}$ and $1 \times 10^{14}\varepsilon$ Hz respectively. Similarly, in the ferroelectric compound KH_2PO_4[9] a value of $f_0 = 5 \times 10^{12}\varepsilon$ Hz has been found.

Lower relaxation rates can be found when coupling to relaxation processes involving movements of groups of atoms in the structure. In YBCO inelastic processes give rise to ultrasound attenuation peaks associated with relaxation rates $\tau^{-1} \approx 10^5$ Hz which appears too slow in comparison to our finding [10, 11]. More specific to a CDW transition is the coupling to discommensuration domains, though this would lead to activated behavior, and result in a very low characteristic frequency (in the range of kHz for $NbSe_2$ [12]).

Another possibility could be that the ultrasound wave destroys the density wave. This could occur if the condition $h\nu \geq 2\Delta(T)$, with $\Delta(T = 0) \approx k_B T_0$ [13]. This condition may be fulfilled very close to the transition, in a range that depends on frequency, but it is unlikely to be the case here, since the width of the peak is almost frequency independent. Also at a given frequency, the peak amplitude does not increase when T_c is reduced.

Other possibilities involve the coupling to collective excitations of the CDW condensates. When the CDW is pinned, we expect both amplitude modes and phase modes to couple to c_{33}. In 1D CDW systems the pinning frequency ω_i

is order of a couple of 10 GHz. This frequency is reduced when the gap is reduced, and when probing close to the transition. For instance in 1D systems the electric field necessary to depin the CDW $E_{\mathrm{T}} \propto \omega_i^2$ decreases exponentially with $T - T_{\mathrm{c}}$ [14].

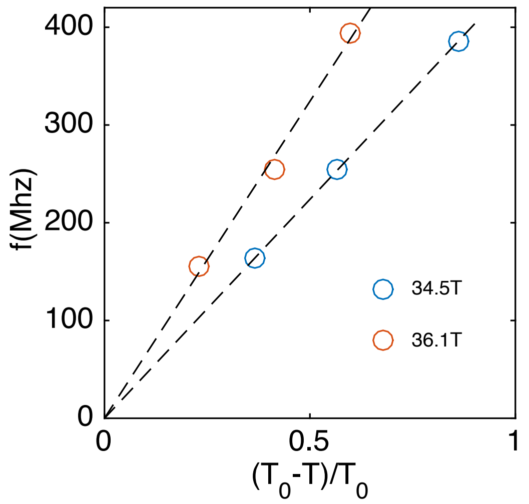

Supplementary Figure 3 Position of the attenuation peak at the α transition as a function of reduced temperature, with $T_0 \approx 5$ K, for two different magnetic fields : 34.5 T in blue and 36.1 T in red. When increasing the frequency, the attenuation peak maximum moves away from the zero frequency transition temperature, as discussed in the main text, following a linear temperature dependence.

Supplementary Note 4 Magnetostriction

The measurement of the sound velocity is performed by measuring the change in the phase of an acoustic wave going through the sample. The measured phase change $\Delta\phi$ is either due to a change of the sample length or in the sound velocity : $\frac{\Delta\phi}{\phi_0}$=-$\frac{\Delta v}{v} + \frac{\Delta L}{L}$. Usually, the length change due to temperature variations (thermal expansion) or due to magnetic field (magnetostriction) can be neglected in comparison to the velocity changes [15]. However, in the case of graphite the large magnetostriction [16] requires a careful inspection of this assumption. For that reason, in addition to the sound velocity measurement reported in the main text, we have measured the magnetostriction.

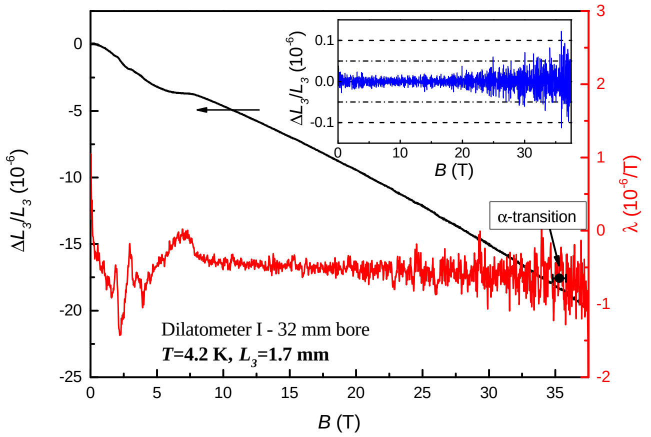

Supplementary Figure 4 c-axis magnetostriction of HOPG graphite at T=4.2K up to 37T.

Magnetostriction measurements were performed using two miniaturized high-resolution capacitance dilatometers [17] at the HFML that fit in either the 32 mm bore magnet up to magnetic fields of 37.5 T or the 50 mm bore magnet up to 30 T at different temperatures. In Supplementary figure 4, we show the c-axis magnetostriction ($\frac{\Delta L_3}{L_3}$) and its derivative $\lambda = \frac{d\frac{\Delta L_3}{L_3}}{dB}$ up to 37.5 T at $T = 4.2$ K. The position of the α-transition is indicated in black points on the Supplementary figure 4. The error bar indicates its variation from sample to sample. At low magnetic fields, we

observe quantum oscillations on the top of monotonic background. Within the accuracy of our measurement ($\frac{\Delta L_3}{L_3} \approx$ 1e-7), we find no anomaly at the α-transition as seen in the inset of the Supplementary figure 4.

We have further measured $\frac{\Delta L_3}{L_3}$ up to 30 T at $T = 0.3$ and 1.8 K, respectively, and present the c-axis magnetostriction in the supplementary figure 5. Again no anomaly is observed within the accuracy of our measurement ($\frac{\Delta L_3}{L_3} \approx$ 5e-8).

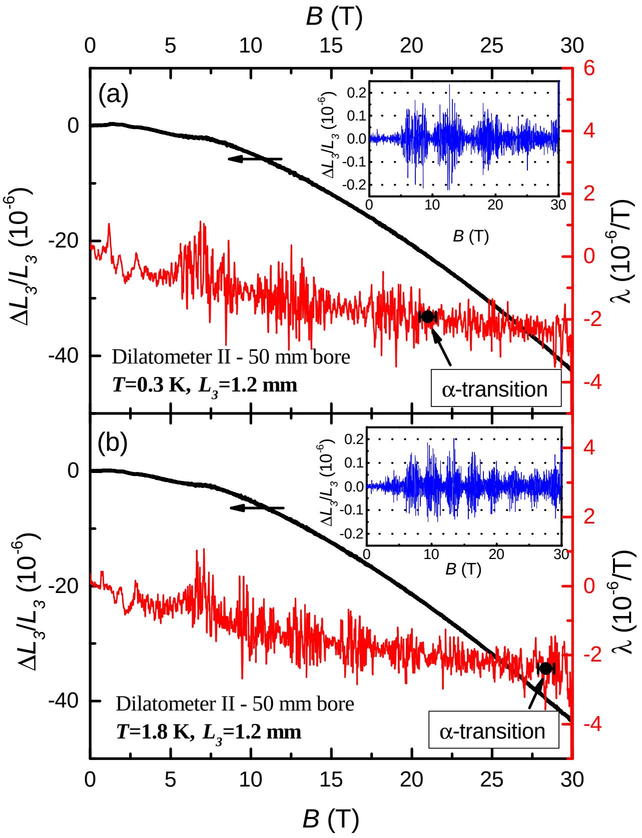

Supplementary Figure 5 c-axis magnetostriction of HOPG graphite (a) $T = 0.3$ K and (b) $T = 1.8$ K up to 30T.

We thus conclude that $\frac{\Delta\phi}{\phi_0}$=-$\frac{\Delta v}{v}$. We can now safely use the sound velocity data in order to perform a thermodynamic analysis. We also show in the next section that the absence of observed anomaly in magnetostriction is consistent with the size of the anomaly in the sound velocity.

Supplementary Note 5 Thermodynamic analysis

Thermodynamic formula - At a second order phase transition the elastic constant $c_{ii} = \frac{1}{V}(\frac{\partial^2 F}{\partial \epsilon_{ii}^2})_T$ with F the free energy and the isobaric specific heat C_p, are interdependent and related through the Ehrenfest relations (see for example [18]). The strain-dependent transition temperature T_c is:

$$\left(\frac{\partial T_c}{\partial \epsilon_i}\right)^2 = \frac{-V_{mol} T_c \Delta c_{ii}}{\Delta C_p(T_c)} \quad (1)$$

with ϵ_i the strain, $V_{\text{mol}} = 5.3 \times 10^{-6}$ m^3.mol^{-1}, T$_c$ the critical temperature and Δc_{ii} and ΔC_p are respectively the jump at T$_c$ in c_{ii} and C_p.

In order to use Supplementary Eq . 1 quantitatively, one must have knowledge of at least two measured quantities in order to deduce a third one. We only measured the jump in the sound velocity, so we must rely on approximations whether for $\frac{\Delta C_p(T_c)}{T_c}$ or for $\frac{\mathrm{d}T_c}{d\varepsilon_i}$.

Estimation of the heat capacity jump at the α-transition

We first use the measured jump in the sound velocity and the available hydrostatic pressure data to estimate what could be the jump in the heat capacity at the α transition. We assume that the hydrostatic pressure dependence is dominated by the c-axis pressure dependence such that

$$\frac{\mathrm{d}T_\mathrm{c}}{dP} = \sum_i \frac{\mathrm{d}T_\mathrm{c}}{dP_i} \approx \frac{\mathrm{d}T_\mathrm{c}}{dP_3} \tag{2}$$

This assumption is supported by two facts. First, the in-plane hopping parameter γ_0 of the SWMc model has a pressure dependence ten-times smaller than the out-of-plane hopping term γ_2 [19], and second, the pressure dependence of γ_2 has been shown to explain the pressure dependence of the α transition temperature [20]. The parameter $\mathrm{d}T_\mathrm{c}/dP$ can be deduced from the study of the pressure dependence of the α-transition, as reported by Iye and al. [20]. According to this work, in the zero pressure limit, $\mathrm{d}T_\mathrm{c}/dP(B)$=$\alpha * (1 - B^*/B_c)T_c$ where α=0.029 $\pm$0.001, B^*=105T. At T=4.2K the critical field is 30T, giving a $\mathrm{d}T_c/dP$=-3e-4K.bar^{-1}=-0.3 K.GPa^{-1}. Then we convert the pressure dependence into a strain dependence:

$$\frac{\mathrm{d}T_\mathrm{c}}{d\varepsilon_3} = \sum_i c_{i3}\frac{\mathrm{d}T_\mathrm{c}}{dP_i} \sim c_{33}\frac{\mathrm{d}T_\mathrm{c}}{dP_3} \sim c_{33}\frac{\mathrm{d}T_\mathrm{c}}{dP} = -11 \text{ K} \tag{3}$$

where we've taken $c_{33} = 36$ GPa [21],

Combining this result with our measurement of the jump of the sound velocity v, $\Delta v/v \sim -2.10^{-5}$, we can estimate the jump of the heat capacity ($\Delta C_p(T_c)$) at the α transition through Supplementary Eq. 1. We find that $\frac{\Delta C_p(T_c)}{T_c} \approx 30$ mJ.K^{-2}.mol^{-1}. This is three orders of magnitude higher than the electronic contribution ($\Delta C_p^{ele} \approx \gamma_e T_\mathrm{c}$) where $\gamma_e = 14$ μJ.K^{-2}.mol^{-1} is the Sommerfeld coefficient of graphite [22, 23]. Note that in presence of a magnetic field a decrease of the Sommerfeld coefficient has been reported which will enhance this difference [24]. This analysis suggests that there is another order parameter, most likely associated with the lattice, that appears at the α transition. The α transition is accompanied by a structural change, that contributes to the change in the total heat capacity and in the sound velocity jump.

Note that this analysis is consistent with the absence of a jump in the magnetostriction. Using the following equation:

$$\frac{\mathrm{d}T_\mathrm{c}}{dP_i} = -\frac{\Delta c_{ii}}{c_{ii}^2 \Delta\lambda_i} \tag{4}$$

with λ_i the thermal expansion coefficient, we estimate the variation of $\frac{\Delta L_{33}}{L_{33}}$ of the order of 5×10^{-7} comparable with the noise level in the magnetostriction experiment.

Estimation of the strain derivative of the critical temperature of the α transition

Applying the Ehrenfest relationship this time to estimate the uniaxial strain dependence, using the measured sound velocity jump and assuming $\Delta C_p(T_\mathrm{c}) = \gamma_e = 13.8$ μ J.K^{-2}.mol^{-1} [24] one obtains $\|\mathrm{d}T_\mathrm{c}/\mathrm{d}\varepsilon_3\| \sim 618$ K or $\|\mathrm{dln}T_\mathrm{c}/\mathrm{d}\varepsilon_3\| \sim 147$ for $T_\mathrm{c} = 4.2$ K. We can estimate the uniaxial pressure dependence from our measured value of $\|\mathrm{d}T_\mathrm{c}/\mathrm{d}\varepsilon_3\|$. Since $s_{13} = s_{23} << s_{33}$

$$\frac{\mathrm{d}T_\mathrm{c}}{dP_3} \approx s_{33}\frac{\mathrm{d}T_\mathrm{c}}{d\varepsilon_3} \tag{5}$$

This yields a large $\frac{\mathrm{d}T_\mathrm{c}}{dP_3} \sim 25$ K/GPa , more than 5 times greater than the out-of-plane pressure dependence of the incommensurate CDW transition in $TaSe_2$ [25]. In this case, the large jump in the sound velocity is explained by a large strain susceptibility of the α transition.

What those two analysis reveal is that the anomaly observed at the onset of the field-induced DW state implies that the lattice is involved in the instabilities found in the quantum limit of graphite whether in the form of a structural transition or in the form of high strain susceptibility of the electronic order parameter. The role of the lattice in the quantum limit of graphite has often been neglected [26], hence this work calls for an examination of the electron-lattice interaction and its role in the mechanism for the DW formation in graphite.

Supplementary References

---

[1] V.L. Gurevich, V.G. Skobov and Yu. A. Firsov, Sov. Phys. JETP, **13**,552 (1961)

[2] T. Inoue, T. Fukami, H. Kuriyaki and S. Mase, Effects of the electron-hole interaction on the giant quantum attenuation and velocity change of sound waves in pyrolytic graphite, Journal of the Physical Society of Japan **52**, 2862-2869

[3] H. Yaguchi, Y. Iye, T. Takamasu and N. Miura, Magnetic-field-induced electronic phase transition in graphite –Pulse field experiment at 3He temperature, Physica B **184**, 332-336 (1993).

[4] F. Arnold, A. Isidori, E. Kampert, B. Yager, M. Eschrig, J. Saunders, Charge density waves in graphite; towards the magnetic ultra-quantum limit, Preprint at http://arxiv.org/abs/1411.3323 (2014)

[5] D. Schoenberg, *Magnetic Oscillations in Metals* , Cambridge University Press (1984)

[6] Zengwei Zhu, Huan Yang, Benot Fauqu, Yakov Kopelevich and Kamran Behnia, Nernst effect and dimensionality in the quantum limit, Nature Phys., **6** 26 (2010).

[7] B. Golding, D. J. Bishop, B. Batlogg, W. H. Haemmerle, Z. Fisk, J. L. Smith, and H. R. Ott, Observation of a collective mode in superconducting UBe_{13}, Phys. Rev. Lett. **55**, 2479 (1969)

[8] P. C. Hohenberg, B. I. Halperin, Theory of dynamic critical phenomena, Rev. Mod. Phys. **49** 435 (1977)

[9] C. W. Garland and D. B. Novotny, Ultrasonic velocity and attenuation in KH_2PO_4, Phys. Rev. **177**, 971 (1969)

[10] D. P. Almond, M. W. Long and G. A. Saunders , Anelastic relaxation evidence of very low activation energy processes in superconducting YBa2Cu3O7-x: magnetic chain excitations?, J. Phys. Cond Matt. **2** 4667 (1990)

[11] J. R. Cooper, J. W. Loram, I. Kokanovi, J. G. Storey, and J. L. Tallon, Pseudogap in $YBa_2Cu_3O_{6+\delta}$ is not bounded by a line of phase transitions: Thermodynamic evidence, Phys. Rev. B **89** 201104 (2014)

[12] M. Barmatz, L. R. Testardi and F.J. Di Salvo, Elasticity measurements in the layered dichalcogenides $TaSe_2$ and $NbSe_2$, Phys. Rev. B **12**, 4367 (1975).

[13] V. M. Bobetic, Evaluation of High-Frequency Ultrasonic Attenuation in Superconductors in the Bardeen-Cooper-Schrieffer Theory of Superconductivity, Phys. Rev. **136**, A1535 (1964)

[14] P. Monceau and G. Gruner, Charge Density Waves in Solids, Modern Problems in Condensed Matter Sciences, Vol. 25

[15] Bruno Lüthi, *Physical acoustics in the solid state*, Springer series in solid-state (2004).

[16] J Heremans, J-P Michenaudt, M Shayegan and G Dresselhaus, Magnetostriction and deformation potentials in graphite, J. Phys. C: Solid State Phys., **14**, 3541-3546 (1981)

[17] R. Kuchler, T. Bauer, M. Brando, and F. Steglich, A compact and miniaturized high resolution capacitance dilatometer for measuring thermal expansion and magnetostriction, Review of Scientifc Instruments **83**, 095102 (2012)

[18] L.R Testardi, Elastic modulus, thermal expansion, and specific heat at a phase transition, Phys. Rev. B, **12**,3849 (1975)

[19] E. Mendez, A. Misu and S. Dresselhaus, Magnetoreflection study of graphite under pressure, Phys. Rev. B **21** 827 (1980)

[20] Y. Iye, C. Murayama, N. Mri, S. Yomo, J. T. Nicholls, and G. Dresselhaus, Effect of pressure on the high-magnetic-field electronic phase transition in graphite, Phys. Rev. B **41** 3249 (1990).

[21] N. B. Brandt, S. M. Chudinov, *Semimetals, Modern Problems in Condensed Matter Sciences*, v: 20.1, Elsevier Science Publishers, (1988)

[22] J. C. van der Hoeven, Jr. and P. H. Keesom, Specific Heat of Various Graphites between 0.4 and 2.0 K, Phys. Rev. **130**, 1318 (1963).

[23] M.G. Alexander, D.P. Goshorn and D.G. Onn, Low-temperature specific heat of the graphite intercalation compounds KC8, CsC8, RbC8, and their parent highly oriented pyrolytic graphite, Phys. Rev. B 22, 4535 (1980)

[24] G. D. Khattak, H. V. Bohm, P. H. Keesom, Anomalous specific heat of pure graphite around 1.45 K in magnetic fields up to 13.7 T, Phys. Rev. B **18**, 6178 (1978).

[25] C. W. Chu, L. R. Testardi, F. J. Di Salvo, and D. E. Moncton, Pressure effects on the charge-density-wave phases in $2HTaSe_2$, Phys. Rev. B, **14**, 464 (1976).

[26] D. Yoshioka and H. Fukuyama, Electronic Phase Transition of Graphite in a Strong Magnetic Field, J. Phys. Soc. Jpn. **50**, 725 (1981).
